# Insights into Chemical Structure-Based Modeling for New Sweetener Discovery

**DOI:** 10.3390/foods12132563

**Published:** 2023-06-30

**Authors:** Ning Tang

**Affiliations:** Beijing Key Laboratory of Functional Food from Plant Resources, College of Food Science and Nutritional Engineering, China Agricultural University, Beijing 100083, China; ningtang@cau.edu.cn; Tel.: +86-010-6273-7401

**Keywords:** machine learning, sweet taste, sweetness, T1R2-T1R3, binding

## Abstract

The search for novel, natural, high-sweetness, low-calorie sweeteners remains open and challenging. In the present study, the structure-based machine learning modeling and sweetness recognition mechanism were investigated to assist this process. It was found that whether or not a compound was sweet was closely related to molecular connectivity and composition (the number of hydrogen bond acceptors and donors), tpsaEfficiency, structural complexity, and shape (nAtomP and Fsp3). While the relative sweetness of sweet compounds was more determined by the molecular properties (tpsaEfficiency and Log P), structural complexity and composition (nAtomP and ATSm 1). The built machine learning models exhibited very good performance for classifying the sweet/non-sweet compounds and predicting the relative sweetness of the compounds. Moreover, a specific binding pocket was found for sweet compounds, and the sweet compounds mainly interacted with the VFT domain of the T1R2-T1R3 through hydrogen bonds. In addition, the results indicated that among the sweet compounds, those that were sweeter bound to the VFT domain stronger than those that had low sweetness. This study provides very useful information for developing new sweeteners.

## 1. Introduction

Sweetness, a receptor-mediated taste, is considered to be the primary determinant of food preference and intake as it produces a pleasant sensation [1]. Therefore, sweet compounds such as sugars or saccharides are widely used in the food industry. These sweet compounds, however, are associated with high calories [2]. The steadily increasing daily sugar consumption, especially in developed countries, will contribute to obesity, type-2 diabetes, and cardiovascular diseases [3]. According to the World Health Organization, the above-mentioned chronic diseases are causing 38 million deaths per year [4]. Low-calorie sweeteners are promising alternatives to replace sugars as they can reduce the calories of sweet foods and beverages while maintaining the high sweetness perception. Since low-calorie is required for both diabetic and dietetic foods, low-calorie sweeteners are highly demanded in the food industry [5,6]. To date, some low-calorie sweeteners have been identified or chemically synthesized and used in the food industry [7]. These obtained low-calorie sweeteners, however, have bitter or metallic off-taste when used in high concentrations [8]. Furthermore, the demand for low-calorie sweeteners, especially natural or food-derived low-calorie sweeteners, has increased rapidly in recent years [5]. Previous studies have outlined key actions proposed in current research related to sweet taste and low-calorie sweeteners. These actions encompass investigating the mechanisms involved in sweetness recognition, discovering novel natural low-calorie sweeteners, assessing the effects of these sweeteners on diverse populations and life stages, and enhancing risk communication and consumer perception. Research has focused on unraveling the intricate processes underlying the perception of sweetness, aiming to understand the molecular and neural mechanisms involved. Furthermore, efforts have been made to identify alternative natural, low-calorie sweeteners that provide a satisfying taste while reducing caloric intake. Accordingly, it is important to investigate the sweetness recognition mechanism and figure out the relationship between the sweetness and the structure of the known sweeteners. This could be very useful for us to identify new low-calorie sweeteners that could be used in the food industry. The search for novel natural or food-derived low-calorie sweeteners, however, remains open and challenging.

The sweet taste is mediated by a T1R2-T1R3 heterodimer that both subunits belong to class C G protein-coupled receptor [9,10]. This sweet taste receptor is expressed at the surface of taste buds and contains three domains: a “Venus Fly Trap” (VFT) domain, a cysteine-rich domain located in the extracellular region, and a transmembrane domain [11]. According to previous studies, the interaction between the sweetener and the extracellular domain of the T1R2-T1R3 is of importance for sweetness recognition [12]. Moreover, some studies suggested that the relative sweetness of the investigated sweeteners correlated with the calculated binding between the sweeteners and human T1R2-T1R3 [4]. However, the experimental 3D structure of the human T1R2-T1R3 sweet taste receptor has not been solved yet. Accordingly, the high-quality homology model of the human T1R2-T1R3 will be very useful for elucidating the mechanism of sweeteners eliciting sweetness. In addition, it is essential to establish a link between the structure information of the known sweeteners and their sweet taste in order to interpret the crucial physico-chemical properties for explaining the sweetness of a molecule. All the above-mentioned information is of importance to design new sweeteners. In the present study, we accordingly collected two databases and then built machine learning models to discriminate sweeteners from non-sweet molecules and to predict the sweetness of the sweeteners. These machine learning models were very useful for us to understand the molecular features underlying sweet taste perception. Furthermore, we built a homology model of the VFT domain of the human T1R2-T1R3 receptor and used this model to investigate the interactions between the sweeteners/non-sweet compounds and the VFT domain. The present study provides very fundamental and useful information for designing new low-calorie sweeteners that could be used in the food industry. 

## 2. Materials and Methods

### 2.1. Dataset Preparation

In the present study, two datasets (listed in the Supporting Information EXCEL files Tables S1 and S2) were collected from the published literature and further used for sweet/non-sweet binary classification (identify sweet compounds from non-sweet compounds) and sweetness (sweet taste intensity) prediction, respectively [13,14]. The dataset used for binary classification contained 649 chemical compounds (435 sweet and 214 non-sweet) associated with names, canonical simplified molecular input line entry system (SMILES) strings, and predominant tastes (sweet/non-sweet) measured through the trained panelists using a sip and spit method. The sweetness prediction dataset (SweetenersDB) consisted of 316 chemical compounds associated with names, canonical SMILES strings, and relative sweetness values in logarithmic scale (base 10) defined as the sweet taste intensity relative to sucrose. 

### 2.2. Calculation of Molecular Fingerprints and Descriptors

The molecular fingerprints were generated with an in-house script based on the rcdk package using the SMILES strings listed in the datasets [15]. The obtained fingerprints were hashed fingerprints (1024 bit) but with the rings and atomic properties taken into account. In addition, the Tanimoto metric was used to generate a pairwise similarity matrix of the investigated compounds according to the obtained hashed fingerprints. The values in the similarity matrix were between 0 and 1 where 0 indicated non-overlapping fingerprints and 1 indicated identical fingerprints. According to the similarity matrix, the distance matrix can be further obtained through the formula: distance = 1 − similarity. Moreover, the descriptors were generated through the same algorithm using the SMILES strings listed in the datasets. Six types (topological, geometrical, hybrid, constitutional, protein, and electronic) of descriptors were generated. Then some of the obtained descriptors were removed if the descriptor was constant, contained value 0 more than 30%, or contained any NA values. The left descriptors were further used for machine learning modeling.

### 2.3. Clustering and Principal Component Analysis

The optimal number of clusters of the investigated chemical compounds was first calculated using the K-means algorithm combined with the gap statistic method based on the generated distance matrix. Then the K-means algorithm was applied using the Euclidean distance measurement method with 2 clusters (sweet/non-sweet) specified to explore the distance distribution between sweet compounds and non-sweet compounds. Furthermore, principal component analysis was applied according to the calculated descriptors to investigate the natural distribution of the sweet/non-sweet compounds in a reduced dimensional space. The obtained PCs could allow us to verify the existence of relationships between the sweet taste and the descriptors in a multidimensional space.

### 2.4. Machine Learning Modeling

The collected datasets were first split into training sets and testing sets with ¾ of data retained for modeling. The 10-fold cross-validation was constructed each time. Regarding the preprocessing, the calculated descriptors were normalized to have a standard deviation of 1 and a mean of 0 with highly correlated descriptors removed. Then, 8 different machine learning algorithms (logistic regression/rule-based regression, decision trees, random forest, multivariate adaptive regression splines, boosted trees, neural network, K-nearest neighbor, and support vector machine) were used for binary classification and sweetness (sweet taste intensity) prediction. The grid search method was used to compute the performance metrics (accuracy, sensitivity, specificity, root mean squared error, the squared correlation between truth and estimate, or mean absolute error) for the predefined tuning hyperparameters that corresponded to models. The predefined parameter grids used for tuning were generated using a space-filling method with a size of 100. For logistic regression modeling, the total amount of regularization and the proportion of L1 regularization were optimized. For decision trees modeling, the maximum depth of the tree and the minimum number of data points in a node that needed to be split further were optimized. For random forest and boosted trees modeling, the number of randomly sampled predictors at each split when creating tree models and the minimum number of data points in a node that needed to be split further were optimized. For multivariate adaptive regression splines modeling, the number of features retained in the final model was optimized with the highest possible interaction degree of 2. For neural network modeling, the number of units in the hidden layer and the amount of weight decay were optimized with the rectified linear unit activation function. This function is an activation between the hidden and output layers. For K-nearest neighbor modeling, the number of neighbors to consider and the number for the parameter used in calculating Minkowski distance were optimized. For support vector machine modeling, the radial basis function was used to create the decision boundary or regression line. The classification models were performed to maximize the width of the margin between classes, while the regression models were performed to optimize a robust loss function. Furthermore, the cost of predicting a sample within or on the wrong side of the margin and the number used for radial basis function were optimized for both classification and regression in support vector machine modeling. For rule-based regression modeling, the number of members of the ensemble and the number of neighbors in the post-model instance-based adjustment set were optimized. After the hyperparameter tuning, the best hyperparameters were selected for the final model fitting according to the collected performance metrics. With the machine learning models built, the best binary classification model was further used to predict the taste class of the chemical compounds in FOODB, the world’s largest and most comprehensive resource on food constituents [16]. As small molecules were considered in the present study, the chemical compounds in FOODB with molecular weight lower than 1000 were selected for taste prediction. The known sweeteners in FOODB but not in our collected datasets were identified by detecting the sweetener keyword in the description provided by the FOODB. 

### 2.5. Homology Modeling and Molecular Docking

As the human T1R2-T1R3 structure is not available yet, the homology modeling was accordingly performed to construct the 3D structure of the VFT domain of the human T1R2-T1R3 receptor using SWISS-MODEL. The canonical amino acid sequences of the VFT domain of the human T1R2-T1R3 receptor were obtained from Q8TE23 and Q7RTX0 reported in the Uniprot database [17]. Then, the obtained amino acid sequences were utilized to search the templates using the hetero-oligomeric protein model building mode. The PDB structure (ID 5X2P) of the ligand-binding domain of the medaka fish taste receptor T1R2a-T1R3 was used as the template to generate the final model [18]. The obtained homology model was then validated through ProSA. After the model validation, the obtained structure was then converted into pdbqt format after the addition of polar hydrogen using AutoDock Tools (Vina) [19]. For the investigated chemical compounds, their 3D sdf format was first generated based on the SMILES strings listed in the collected datasets and then converted into pdbqt format through Openbabel. Subsequently, the docking was performed in a predefined grid box (80 × 70 × 80) with an exhaustiveness of 24. The binding sites with the lowest docking score were further collected using an in-house bash script. 

### 2.6. Interactions between the Investigated Chemical Compounds and VFT Domain of T1R2-T1R3

Each VFT domain-ligand complex structure was constructed based on the ligand’s best predicted binding site (the one with the best docking score) obtained from the docking. After the complex construction, each complex was further subjected to the protein –ligand interaction profiler (PLIP) analysis [20]. The hydrogen bonds, hydrophobic contacts, π-stacking, π-cation interactions, salt bridges, water bridges, and halogen bonds were calculated. In addition, the results were collected in XML files and used for further analysis.

## 3. Results and Discussion

### 3.1. Similarity of the Sweet and Non-Sweet Chemical Compounds

**Figure 1:**
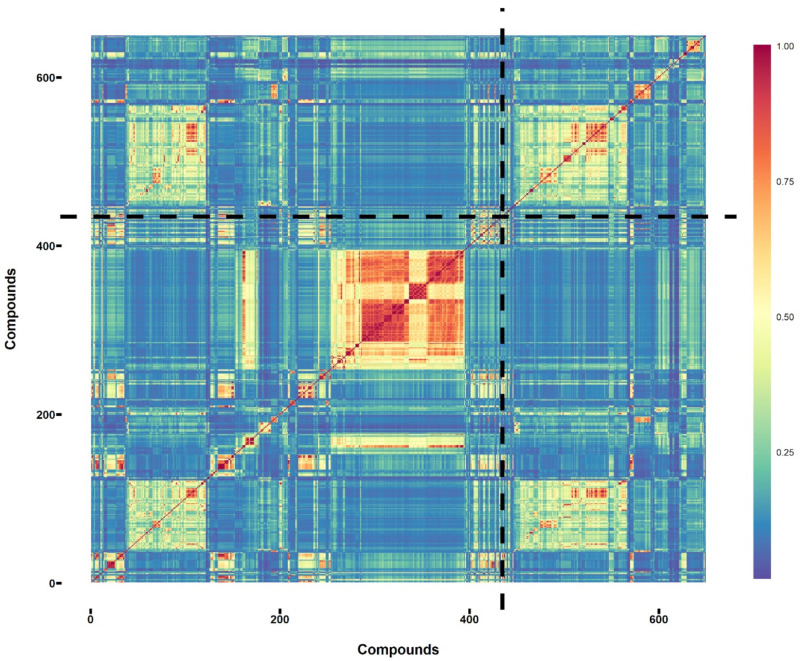
Similarity matrix of the sweet/non sweet compounds used for binary classification (Table S1) calculated based on Tanimoto correlation coefficient. The sweet and non-sweet compounds were separated by the dash lines (the first 435 compounds were sweet, and the other 214 compounds were non-sweet).

Based on the similarity property principle, molecules with similar molecular structures are more likely to have similar properties [21]. Accordingly, if some similarity patterns were found for the sweet compounds, it would be possible to identify the sweet compounds located at a certain distance from the known sweeteners. In order to investigate the similarity of our investigated chemical compounds, the Tanimoto coefficients were calculated using the following formula according to the generated fingerprints:

S_A,B_ = c/(a + b − c) (1)

where S is the similarity, a is the number of fingerprint bits in molecule A, b is the number of fingerprint bits in molecule B, and c is the number of fingerprint bits in both molecules [22]. The obtained similarity matrix is shown in Figure 1 with the sweet and non-sweet chemical compounds separated by the dashed lines. As can be seen from Figure 1, although some warm-color blocks were found in the sweet compounds versus sweet compounds region of the heatmap, the average Tanimoto coefficient between all the investigated sweet compounds was 0.246, indicating that the differences between the investigated sweet compounds were large. The differences between the sweet and non-sweet compounds were larger, as indicated by the average Tanimoto coefficients of 0.178, suggesting the calculated fingerprints could capture some differences between sweet and non-sweet compounds. Some warm-color blocks, however, could also be noted in the sweet compounds vs. non-sweet compounds region of the heatmap. This suggested that it could be difficult to successfully distinguish the sweet/non-sweet compounds only using the similarity information. Furthermore, the K-means clustering, a widely used clustering method, showed a similar result when using the calculated distance matrix as the input. As can be seen from Figure S1, the optimal number of clusters was seven according to the gap statistic calculation. If two clusters (sweet/non-sweet) were specified in K-means clustering modeling (Figure S2), those in obtained cluster one were all sweet compounds, again indicating the difficulty of distinguishing sweet/non-sweet compounds simply based on the similarity distance. 

### 3.2. Principal Components Analysis (PCA) and Receiver Operating Curve (ROC) Analysis

Accordingly, six types of molecular descriptors were further calculated to obtain a better classification of the sweet/non-sweet compounds. Based on the rcdk package, 287 molecular descriptors were obtained, and 91 molecular descriptors were retained after the preprocessing. Then, dimension reduction was performed using PCA, and the results are shown in Figures S3 (score plot) and S4 (loading plot). As can be seen from Figure S3, the PC1 (68.1%) and PC2 (10.7%) together explained the 78.8% variation in the investigated data set, but it was very clear that the dimension reduction method could not separate the sweet/non-sweet compounds. Most of the non-sweet compounds were located in the second quadrant with most of the PC1 values close to 0 and positive PC2 values. According to the loading plot shown in Figure S4, the non-sweet compounds were more characterized by the Log P values, while the sweet compounds were more characterized by Log P values and tpsaEfficiency. This was in agreement with previous drug design studies, suggesting that the Log P value has higher importance than that of the other descriptors [23]. In addition, the number of hydrogen bond donors and acceptors also contributed significantly to the dimension reduction. Furthermore, we performed an ROC analysis using a single descriptor as the classifier to distinguish the sweet/non-sweet compounds, and the results are shown in Figure S5. As can be seen from Figure S5, some of the descriptors performed reasonably well in distinguishing the sweet/non-sweet compounds, with the AUC values being around 0.78. The top classifiers were TopoPSA, nHBDon, nHBAcc, and tpsaEfficiency, respectively. Compared with the PCA results, the ROC analysis demonstrated better classification ability, indicating that descriptors with higher AUC values were relatively more important features that contributed to the sweet or non-sweet taste. 

### 3.3. Machine Learning Models

**Figure 2:**
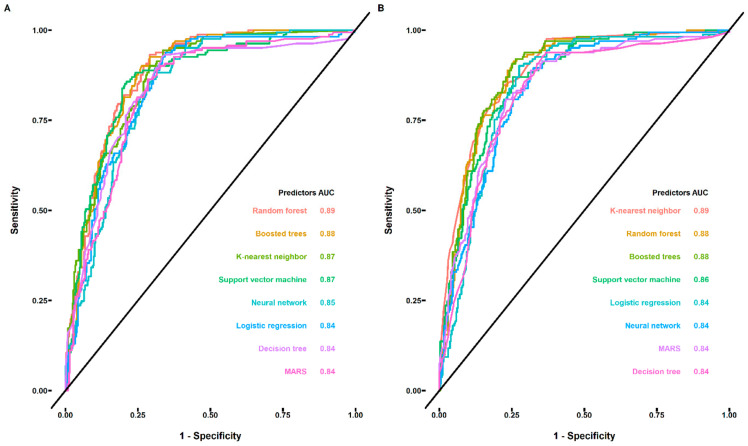
The receiver operating characteristic (ROC) curve analysis for evaluating the performance of the built machine learning classification models (logistic regression, decision trees, random forest, multivariate adaptive regression splines, boosted trees, neural network, K-nearest neighbor, and support vector machine). The area under curve (AUC) values listed in each figure were calculated based on the unsmoothed ROC curve. (**A**) The ROC curve for the built machine learning classification models with 91 descriptors as the input. (**B**) The ROC curve for the built machine learning classification models with top 10 descriptors selected through the variable importance analysis as the input.

The above results clearly indicated that it was quite difficult to successfully distinguish the sweet and non-sweet compounds by simply relying on the calculated descriptors. Accordingly, we built eight machine learning models to predict taste class and sweetness (sweet taste intensity). For classification, logistic regression, decision trees, random forest, multivariate adaptive regression splines boosted trees, neural network, K-nearest neighbor, and support vector machine methods were applied using the retained 91 descriptors as input, and the results are shown in Figure 2A. As can be seen from Figure 2A and Table S3, the performance of all the built models was reasonably good, the random forest and boosted trees were relatively better at distinguishing the sweet/non-sweet compounds as indicated by the AUC values (0.89 and 0.88, respectively), demonstrating the potential power of the ensemble learning methods. In addition, the identified best hyperparameters of each model were also shown in Table S3. For the best classification model (random forest), the number of variables available for splitting at each tree node (mtry) and the number of trees (trees) were identified to be 3 and 1200, respectively. Moreover, the random forest model was further used to calculate the variable importance, and the top 20 descriptors are shown in Figure S6A. The number of hydrogen bond acceptors and donors, tpsaEfficiency, and the number of atoms in the largest pi chain (nAtomP) were more important features for distinguishing the sweet/non-sweet compounds. According to the calculated variable importance, the top 10 descriptors were selected for comparison, and the results are shown in Figure S7. As can be seen from Figure S7, the sweet compounds had more hydrogen bond acceptors and donors and higher tpsaEfficiency but lower nAtomP and Log P values. In addition, the top 10 descriptors were used instead of all 91 to build the machine learning models again. Figure 2B shows the performance of the built models using only 10 descriptors as the input. Notably, the performance was as good as the built models shown in Figure 2A, indicating these 10 descriptors could determine the sweet/non-sweet taste of the compounds very well. In agreement with the models built using 91 descriptors, K-nearest neighbor, random forest, and boosted trees were top models with the AUC values of 0.89, 0.88, and 0.88, respectively. The best hyperparameters of the models are shown in Table S3. For the best model (K-nearest neighbor), the optimal number of neighbors and distance calculation methods were identified to be five and the Manhattan distance method (dist_power = 0.95). Furthermore, to better validate the built models we performed the external validation using the compounds in FOODB that are known to be a sweetener but are not contained in the dataset used for classification modeling. The results showed that both random forest (best model built using 91 descriptors) and K-nearest neighbor (best model built using 10 descriptors) could predict the known sweeteners into sweet class with 97% accuracy (except one artificial sweetener, betamipron) demonstrating the strong predictive power of the built models. Accordingly, the above-built models could be used for identifying sweeteners in food sources, which are of great importance to the food industry.

**Figure 3:**
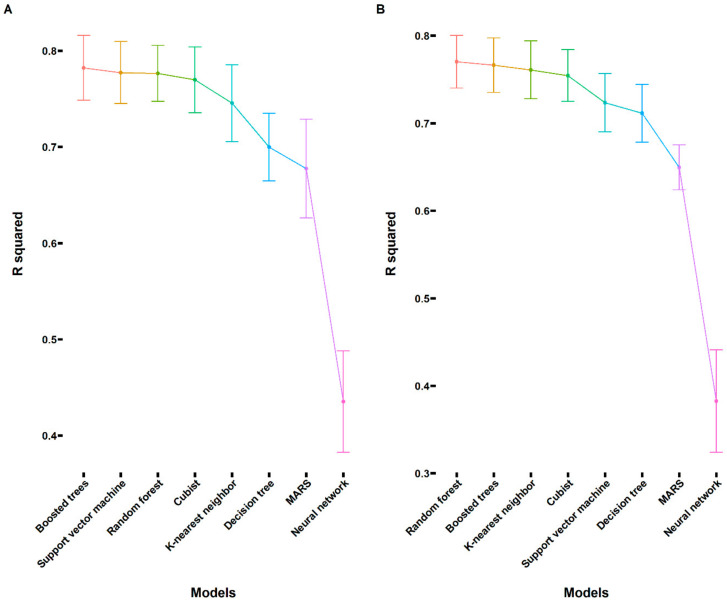
The adjusted R^2^ values for evaluating the performance of the built machine learning regression models (cubist, decision trees, random forest, multivariate adaptive regression splines, boosted trees, neural network, K-nearest neighbor, and support vector machine). (**A**) The adjusted R^2^ values for the built machine learning classification models with 91 descriptors as the input. (**B**) The adjusted R^2^ values for the built machine learning classification models with top 10 descriptors selected through the variable importance analysis as the input.

Besides the sweet taste, the sweetness (sweet taste intensity) of the compounds is also fundamentally important to the food industry and human health. High-sweetness, low-calorie sweeteners are, in general, desired to replace existing sweeteners. It is necessary to investigate the relationship between the sweetness and the structures of the known sweeteners, which will help us to predict the sweetness of a compound. Therefore, we collected another dataset shown in Table S2 (Excel file) with known sweetness to study this relationship. For this dataset, we also performed the PCA first, and the results are shown in Figures S8 and S9. The first two principles explained 83.4% of the variance, and there was good separation between the low-sweetness compounds and other compounds. These low-sweetness compounds were more characterized by the tpsaEfficiency, nHBDon, and TopoSA as illustrated in Figure S9. In addition, the Log Sw was more correlated with the Log P and nAtomP. For sweetness prediction, rule-based regression (Cubist), decision trees, random forest, multivariate adaptive regression splines (MARS), boosted trees, neural network, K-nearest neighbor, and support vector machine methods were used to predict the sweetness with 91 descriptors as the input. As can be seen from Figure 3A, the performance of the built models was quite different according to the evaluation metric adjusted R^2^. As illustrated in Figure 3A, the top three models were boosted trees, support vector machine, and random forest, with an adjusted R^2^ of 0.78 (Table S4), while the neural network exhibited the worst performance, with an adjusted R^2^ of 0.44, indicating the difficulty of sweetness prediction. For the best model (boosted trees), tree depth, the number of predictors that will be randomly sampled at each split (mtry), the number of trees, and learn rate were identified to be 15, 12, 498, and 0.039, respectively (Table S4). Furthermore, we also calculated the variable importance, and the results are shown in Figure S6B. For sweetness prediction, the tpsaEfficiency and Log P values contributed more significantly to predicting the sweetness, which was in agreement with the PCA results. In accordance with the classification modeling, the top 10 descriptors were further selected for building the machine learning models again. Figure 3B shows the performance of the built models with 10 descriptors as the input. Although the adjusted R^2^ exhibited a slight decrease, the best model (random forest) showed an adjusted R^2^ of 0.77, indicating that the sweetness of the compounds could be determined very well by these 10 descriptors. The best hyperparameters for this model were identified to be 2 (mtry) and 101 (trees), respectively. Figures S10 and S11 illustrate the comparison between the predicted and experimental Log Sw values for both models (Figure S10 91 descriptors and Figure S11 10 descriptors). As can be seen from Figures S10 and S11, it was very clear that models were less accurate for high sweetness values since all the models were trained with less information for highly potent sweeteners. Similar results have been reported in previous studies [6]. Accordingly, the quality of the built models could be improved by expanding the chemical diversity of sweet compounds in the dataset. Notably, only structural properties of molecules were considered in the above machine learning modeling. However, the built machine learning models were quite good at predicting the sweetness considering the complexity of the sweetness. This clearly indicated that identified top descriptors during the modeling were important features affecting the sweetness of the compounds. These features could be used for designing new sweeteners. Furthermore, compared to previously established machine learning models [13], our model only utilized descriptors obtained from an open-source cheminformatics package. This approach ensured the future use and prediction of sweet taste or sweetness to be more convenient and standardized.

### 3.4. Interactions between Sweet/Non-Sweet Compounds and Sweet Taste Receptor 

**Figure 4:**
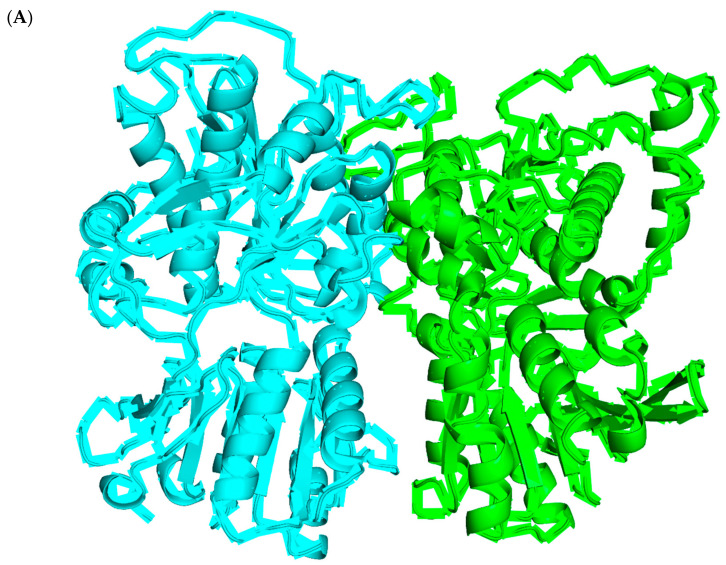

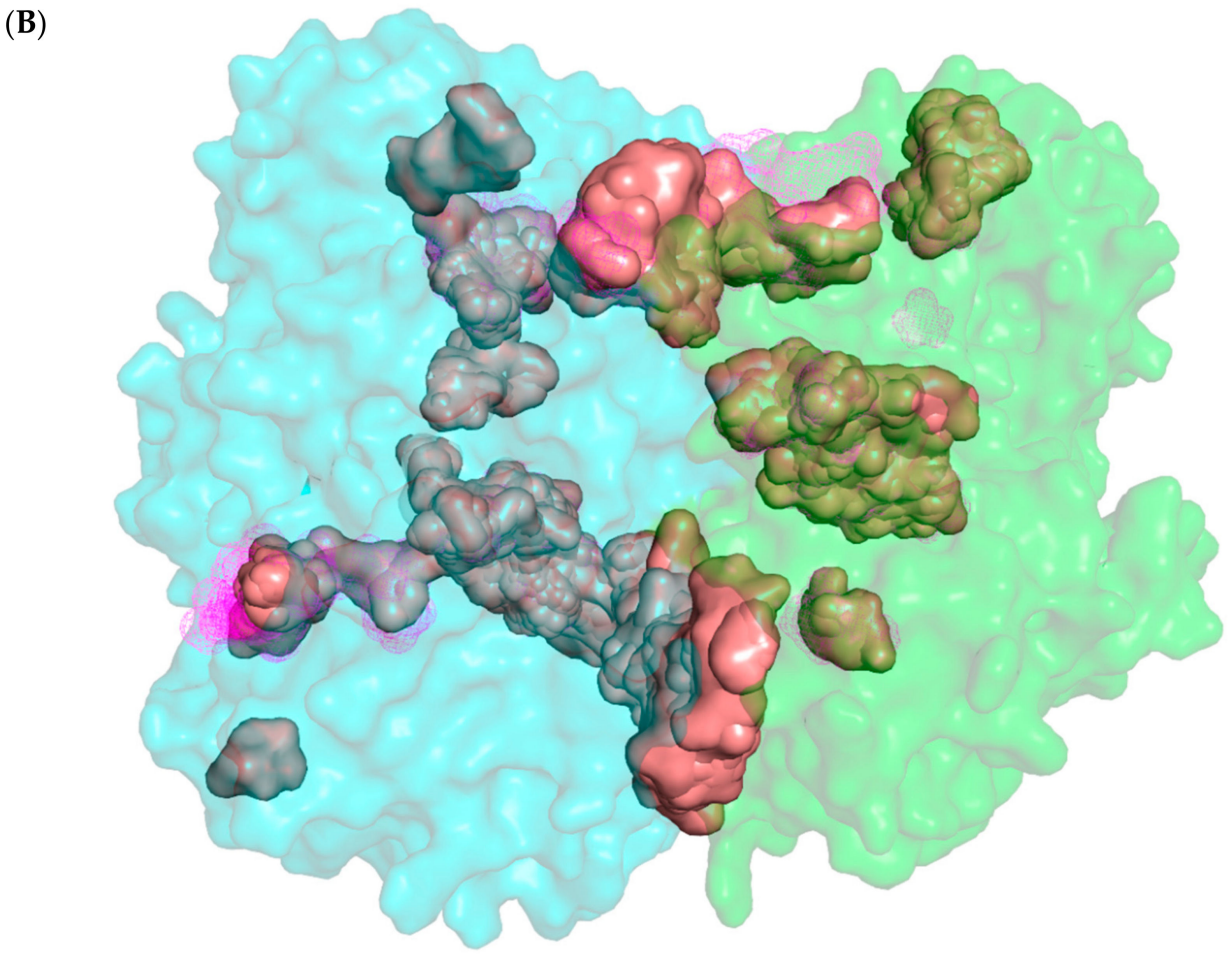
(**A**) The homology model of the “Venus Fly Trap” (VFT) domain of the human sweet taste receptor T1R2-T1R3. The cyan color represents the VFT domain of the T1R2 and the green color represents the VFT domain of T1R3. (**B**) All the binding sites of the sweet and non-sweet compounds (Table S1) in the VFT domain of T1R2-T1R3. The pink surface and magenta mesh represent all the binding sites of sweet and non-sweet compounds in the VFT domain of the T1R2-T1R3, respectively.

**Figure 5:**
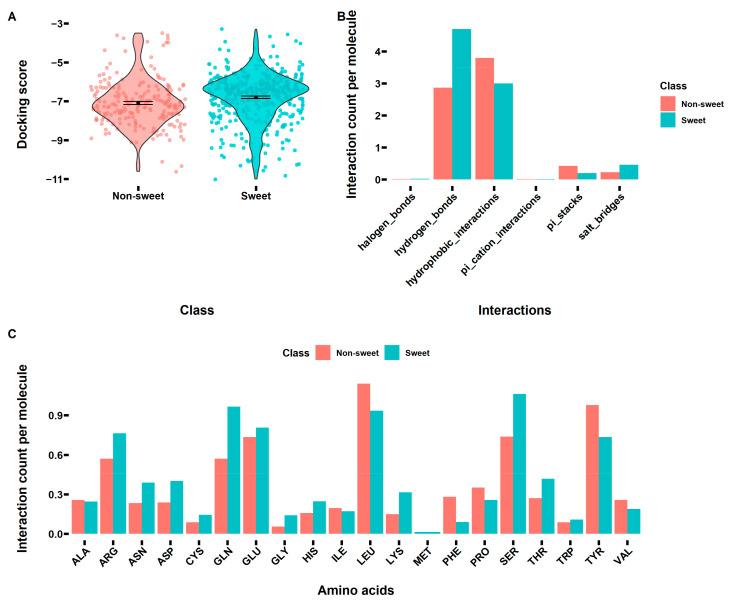
The interactions between the sweet/non-sweet compounds (Table S1) and the VFT domain of T1R2-T1R3. (**A**) The docking scores of the sweet/non-sweet compounds (Table S1). (**B**) The interaction (hydrogen bonds, hydrophobic contacts, π-stacking, π-cation interactions, salt bridges, water bridges, and halogen bonds) counts per molecule between the sweet/non-sweet compounds (Table S1) and the VFT domain of T1R2-T1R3. (**C**) The interaction counts per molecule of the involved amino acids in the VFT domain of T1R2-T1R3.

**Figure 6:**
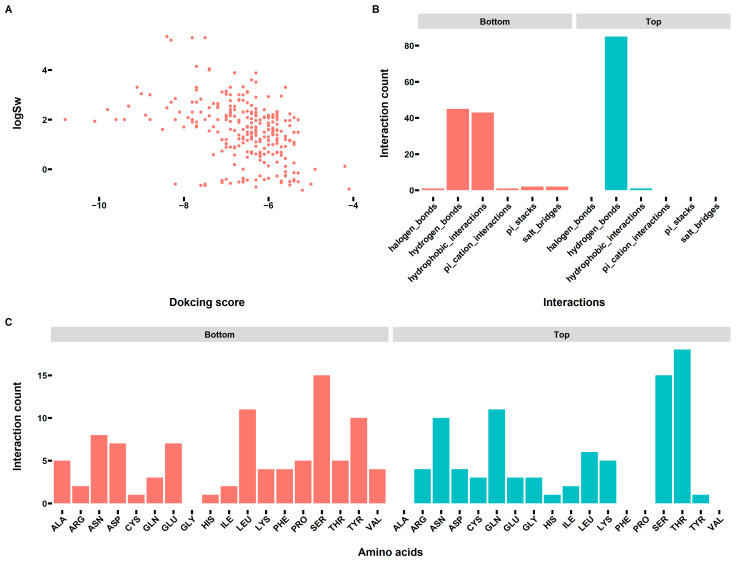
The interactions between the sweet compounds (Table S2) and the VFT domain of T1R2-T1R3. (**A**) The correlation between the sweetness of the investigated compounds (Table S2) and docking scores. (**B**) The interaction (hydrogen bonds, hydrophobic contacts, π-stacking, π-cation interactions, salt bridges, water bridges, and halogen bonds) counts per molecule between the top 10 and bottom 10 compounds (Table S2) and the VFT domain of T1R2-T1R3. (**C**) The interaction counts per molecule of the involved amino acids in the VFT domain of T1R2-T1R3 for the top 10 and bottom 10 compounds.

As mentioned before, eliciting a sweet taste is a very complicated process. Other than the molecular structures of the sweet compounds playing an important role in this process, the interactions between the sweet compounds and the sweet taste receptor should be considered for understanding this process. Previous studies suggested the sweet compounds mainly interacted with the VFT domain of the sweet taste receptor T1R2-T1R3. However, the 3D structure of the human T1R2-T1R3 receptor is not available yet, limiting any further investigation of the interactions. Accordingly, we built a homology model of the VFT domain, and the results are shown in Figure 4A. Then, a z-score was calculated to check the overall quality of the obtained homology model. As can be seen from Figure S12, the z-score for the VFT domain of T1R2 and T1R3 was −9.34 and −8.63, respectively, and both values were within the range of scores typically found for native proteins of similar size, indicating the obtained structure of VFT domain of T1R2-T1R3 was valid and could be used for further investigation. Then, the molecular docking was performed for all the molecules in both datasets. After the docking, the interactions were further analyzed, and the results are shown in Figure 4, Figure 5 and Figure 6. Figure 4B shows binding sites of all the sweet/non-sweet molecules (classification dataset) in the VFT domain of T1R2-TIR3. Although there were some overlap binding sites for both sweet and non-sweet compounds, indicating the common binding pocket for small molecules, the results clearly suggested there was a binding site (pink surface), a cavity formed between the VFT domain of T1R2 and T1R3 only suitable for sweet compounds as no non-sweet compounds were found to be able to bind to this site. This finding suggested that this binding site is of great importance for designing new sweeteners in the future. In addition, for the classification dataset, we did not observe any docking score difference between the sweet and non-sweet compounds (Figure 5A). As shown in Figure 5B, the sweet compounds formed more hydrogen bonds with the VFT domain of T1R2-T1R3, while the non-sweet compounds had more hydrophobic interactions with the VFT domain of T1R2-T1R3. This result was in accordance with the variable importance calculation and ROC analysis (Figure S5), showing that the number of hydrogen bond acceptors and donors was an important feature for determining sweet taste. Moreover, Figure 5C demonstrates that the main residues involved in the interactions were identified to be Arg, Gln, Glu, Leu, Ser, and Tyr, but the sweet compounds had more interactions with the charged residues (Arg, Gln, and Glu) and Ser, which was in agreement with the hydrogen bonds formation analysis. For sweet compounds with known Log Sw values, we observed that the Log Sw values negatively correlated with docking scores, although there were some outliers indicating that the sweeter compounds have relatively higher binding affinity to the VFT domain of T1R2-T1R3 (Figure 6A). Previous studies (a smaller-scale dataset containing only 25 compounds) also suggested that the calculated ΔG_binding_ negatively correlated with the sweetness intensities in different sweetener families [4]. In the present study, we confirmed this trend but found a relatively poor correlation as the compounds were not grouped based on their structural features. Furthermore, the interaction analysis shown in Figure 6B illustrated that sweeter compounds formed more hydrogen bonds with the VFT domain, while less sweet compounds interacted with the VFT domain through hydrogen bonds and hydrophobic interactions, which confirmed the previous results. The residues of the VFT domain involved in the interactions were in general agreement with previously identified residues (Figure 6C).

## 4. Conclusions

In conclusion, we have shown that the built machine learning models exhibited good performance for distinguishing the sweet compounds from non-sweet compounds and predicting the sweetness as indicated by the AUC values and adjusted R^2^. In particular, the best models, which were random forest and boosted trees, showed an AUC value of 0.89 and an adjusted R^2^ of 0.78, respectively. In addition, according to the calculated variable importance, the sweet taste was more determined by the number of hydrogen bond acceptors and donors, tpsaEfficiency, nAtomP, and Fsp3, while the sweetness was more determined by tpsaEfficiency, Log P, nAtomP, and ATSm 1. Considering the interactions between the sweet compounds and sweet taste receptor T1R2-T1R3, it was found that the sweet compounds formed more hydrogen bonds with the VFT domain, while the non-sweet compounds or less sweet compounds interacted with the VFT domain through the hydrophobic interactions and hydrogen bonds. Furthermore, we found that only sweet compounds could bind to a specific binding pocket, a cavity formed between T1R2 and T1R3. This binding pocket will play an important role in new sweetener design. Moreover, the correlation between the docking score and sweetness indicated that sweeter compounds had stronger VFT domain binding affinities, but this correlation needs to be further investigated. The above results provide very useful information for identifying and designing new sweeteners in the future.

## Data Availability

Data is contained within the article or Supplementary Materials.

## Supplementary Materials

The following supporting information can be downloaded at: https://www.mdpi.com/article/10.3390/foods12132563/s1, Figure S1: The calculated optimal number of clusters of the sweet/non-sweet compounds (Table S1) using the gap statistic method. The dash line indicates the optimal number of clusters; Figure S2: The cluster plot of the sweet/non-sweet compounds (Table S1) using k-means clustering method with k = 2 specified in the calculation; Figure S3: The score plot of the principal components analysis (PCA). The calculated coordinates for two groups (sweet/non-sweet) of compounds (Table S1). The coordinates for a given group was calculated as the mean coordinates of the individuals in the group. The data was centered and scaled before the calculation; Figure S4: The loading plot of the principal components analysis (PCA). The calculated coordinates for the properties of sweet/non-sweet compounds (Table S1); Figure S5: The receiver operating characteristic (ROC) curve analysis for evaluating the performance of the individual descriptor as the classifier. The plot shows the performance of the top 20 descriptors according to the obtained area under curve (AUC) values. The AUC values listed in the plot were calculated based on the unsmoothed roc curve; Figure S6: The variable importance of the best machine learning models built with 91 descriptors as the input. (A) The variable importance (top 20 descriptors) of best machine learning classification model (random forest) built with 91 descriptors as the input. (B) The variable importance (top 20 descriptors) of best machine learning regression model (boosted trees) built with 91 descriptors as the input; Figure S7: The value distribution of the top 10 descriptors obtained from the variable importance calculation for the sweet and non-sweet compounds (Table S1); Figure S8: The score plot of the principal components analysis (PCA). The calculated coordinates for the compounds used for the regression modeling (Table S2). The data was centered and scaled before the calculation.; Figure S9: The loading plot of the principal components analysis (PCA). The calculated coordinates for the properties of sweet compounds used for the regression modeling (Table S2); Figure S10: The differences between the log Sw values of the sweet compounds (Table S2) and predicted log Sw values obtained from the machine learning regression models built with 91 descriptors as the input. The solid line in each figure represents the linear regression; Figure S11: The differences between the log Sw values of the sweet compounds (Table S2) and predicted log Sw values obtained from the machine learning regression models built with 10 descriptors (top 10 descriptors obtained from the variable importance calculation) as the input. The solid line in each figure represents the linear regression.; Figure S12: Z scores (overall model quality) of the homology model of the VFT domain of human taste receptor (A) T1R2 and (B) T1R3 and all experimentally determined protein chains in current PDB database. The black dots indicate our obtained homology models; Table S1: Excel file; Table S2: Excel file; Table S3: The performance of the built machine learning classification models (logistic regression, decision trees, random forest, multivariate adaptive regression splines, boosted trees, neural network, K-nearest neighbor, and support vector machine) and best tuned hyperparameters for each model. The first 8 rows were the results for the machine learning models built with 91 descriptors as the input. The following 8 rows were the results for the machine learning models built with 10 descriptors (obtained from the variable importance calculation) as the input; Table S4: The performance of the built machine learning regression models (cubist, decision trees, random forest, multivariate adaptive regression splines, boosted trees, neural network, K-nearest neighbor, and support vector machine) and best tuned hyperparameters for each model. The first 8 rows were the results for the machine learning models built with 91 descriptors as the input. The following 8 rows were the results for the machine learning models built with 10 descriptors (obtained from the variable importance calculation) as the input.
