# Supplementary material for: Insights into Chemical Structure-Based Modeling for New Sweetener Discovery
Source: Foods. 2023 Jun 30;12(13):2563. doi: 10.3390/foods12132563 (PMC10340280; doi:10.3390/foods12132563)
Supplement: Supplementary file 1 [file foods-12-02563-s001.zip › foods-2444588 - for publish suppl words.pdf]

Supporting Information

Insights into chemical structure-based modeling for new sweetener discovery

Ning Tang [*]

Beijing Key Laboratory of Functional Food from Plant Resources, College of Food Science and Nutritional Engineering, China Agricultural University, Beijing 100083, China

*Corresponding authors:

Ning Tang, Tel: +86-010-62737401, E-mail: ningtang@cau.edu.cn

Figure Captions

Figure S1 The calculated optimal number of clusters of the sweet/non-sweet compounds **(Table S1)** using the gap statistic method. The dash line indicates the optimal number of clusters.

Figure S2 The cluster plot of the sweet/non-sweet compounds **(Table S1)** using k-means clustering method with k = 2 specified in the calculation.

Figure S3 The score plot of the principal components analysis (PCA). The calculated coordinates for two groups (sweet/non-sweet) of compounds **(Table S1)**. The coordinates for a given group was calculated as the mean coordinates of the individuals in the group. The data was centered and scaled before the calculation.

Figure S4 The loading plot of the principal components analysis (PCA). The calculated coordinates for the properties of sweet/non-sweet compounds **(Table S1)**.

Figure S5 The receiver operating characteristic (ROC) curve analysis for evaluating the performance of the individual descriptor as the classifier. The plot shows the performance of the top 20 descriptors according to the obtained area under curve (AUC) values. The AUC values listed in the plot were calculated based on the unsmoothed roc curve.

Figure S6 The variable importance of the best machine learning models built with 91 descriptors as the input. **(A)** The variable importance (top 20 descriptors) of best machine learning classification model (random forest) built with 91 descriptors as the input. **(B)** The variable importance (top 20 descriptors) of best machine learning regression model (boosted trees) built with 91 descriptors as the input.

Figure S7 The value distribution of the top 10 descriptors obtained from the variable importance calculation for the sweet and non-sweet compounds **(Table S1)**.

Figure S8 The score plot of the principal components analysis (PCA). The calculated coordinates for the compounds used for the regression modeling **(Table S2)**. The data was centered and scaled before the

calculation.

Figure S9 The loading plot of the principal components analysis (PCA). The calculated coordinates for the properties of sweet compounds used for the regression modeling **(Table S2)**.

Figure S10 The differences between the log Sw values of the sweet compounds **(Table S2)** and predicted log Sw values obtained from the machine learning regression models built with 91 descriptors as the input. The solid line in each figure represents the linear regression.

Figure S11 The differences between the log Sw values of the sweet compounds **(Table S2)** and predicted log Sw values obtained from the machine learning regression models built with 10 descriptors (top 10 descriptors obtained from the variable importance calculation) as the input. The solid line in each figure represents the linear regression.

Figure S12 Z scores (overall model quality) of the homology model of the VFT domain of human taste receptor **(A)** T1R2 and **(B)** T1R3 and all experimentally determined protein chains in current PDB database. The black dots indicate our obtained homology models.

Table S3 The performance of the built machine learning classification models (logistic regression, decision trees, random forest, multivariate adaptive regression splines, boosted trees, neural network, K-nearest neighbor, and support vector machine) and best tuned hyperparameters for each model. The first 8 rows were the results for the machine learning models built with 91 descriptors as the input. The following 8 rows were the results for the machine learning models built with 10 descriptors (obtained from the variable importance calculation) as the input.

Models	penalty	mixture	mean	std_err	tree_depth	min_n	mtry	trees	num_terms	learn_rate	hidden_units	neighbors	dist_power	cost	rbf_sigma
LR	0.0036939	0.375	0.844	0.015											
DT			0.839	0.013	11	13									
RF			0.885	0.014			3	1200							
MARS			0.835	0.015					5						
BT			0.883	0.013	15		7	521		4.97E-08					
NN	1.57E-06		0.847	0.016							6				
KNN			0.869	0.016								14	1.031		
SVM			0.865	0.012										9.11	0.036
LR	1.15E-06	0.987	0.843	0.019											
DT			0.837	0.015	13	21									
RF			0.884	0.014			2	707							
MARS			0.839	0.014					5						
BT			0.883	0.015	10		5	1288		0.000294					
NN	0.0002055		0.842	0.018							8				
KNN			0.888	0.016								5	0.945		
SVM			0.863	0.019										20.3	0.054

Table S4 The performance of the built machine learning regression models (cubist, decision trees, random forest, multivariate adaptive regression splines, boosted trees, neural network, K-nearest neighbor, and support vector machine) and best tuned hyperparameters for each model. The first 8 rows were the results for the machine learning models built with 91 descriptors as the input. The following 8 rows were the results for the machine learning models built with 10 descriptors (obtained from the variable importance calculation) as the input.

Models	committees	tree_depth	min_n	mean	std_err	mtry	trees	num_terms	learn_rate	hidden_units	penalty	neighbors	dist_power	cost	rbf_sigma
Cubist	35			0.770	0.034							10			
DT		8	26	0.700	0.035										
RF				0.776	0.029	3	1813								
MARS				0.678	0.051			5							
BT		15		0.782	0.034	12	498		0.039						
NN				0.435	0.053					8	4.03E-05				
KNN				0.746	0.040							10	1.506		
SVM				0.777	0.032									19.055	0.054
Cubist	20			0.754	0.030							5			
DT		13	28	0.711	0.033										
RF				0.770	0.030	2	101								
MARS				0.650	0.026			4							
BT		4		0.766	0.031	4	391		0.068						
NN				0.382	0.059					8	7.36E-05				
KNN				0.761	0.033							6	0.103		
SVM				0.723	0.033									0.796	0.047

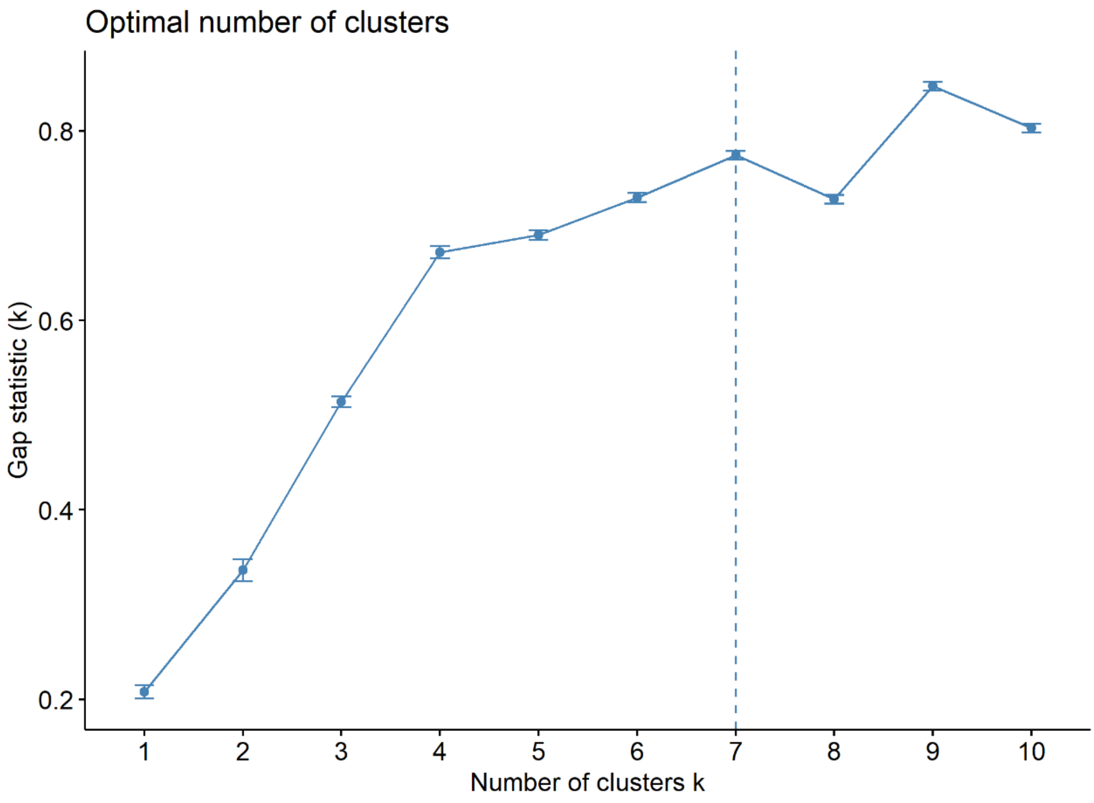
Optimal number of clusters
0.8
0.6
0.4
0.2
Gap statistic (k)
1
2
3
4
5
6
7
8
9
10
Number of clusters k

Figure S1

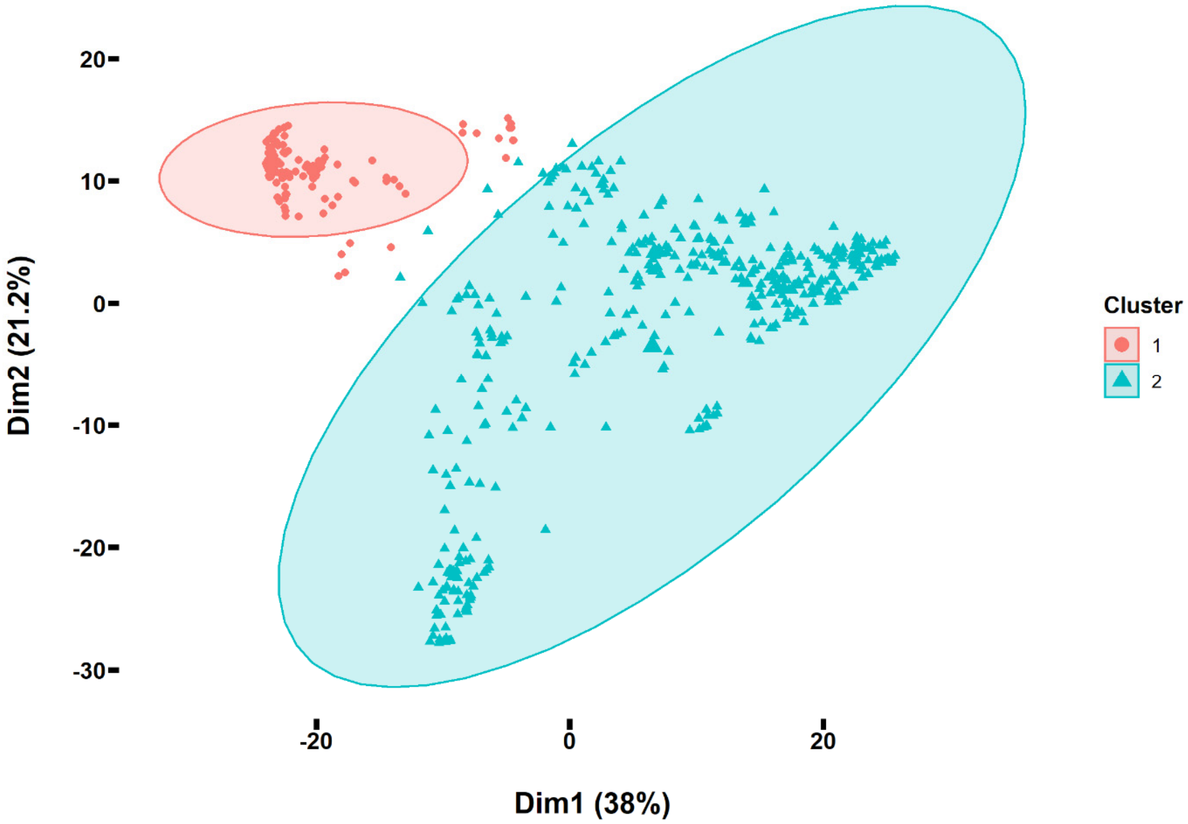

Figure S2

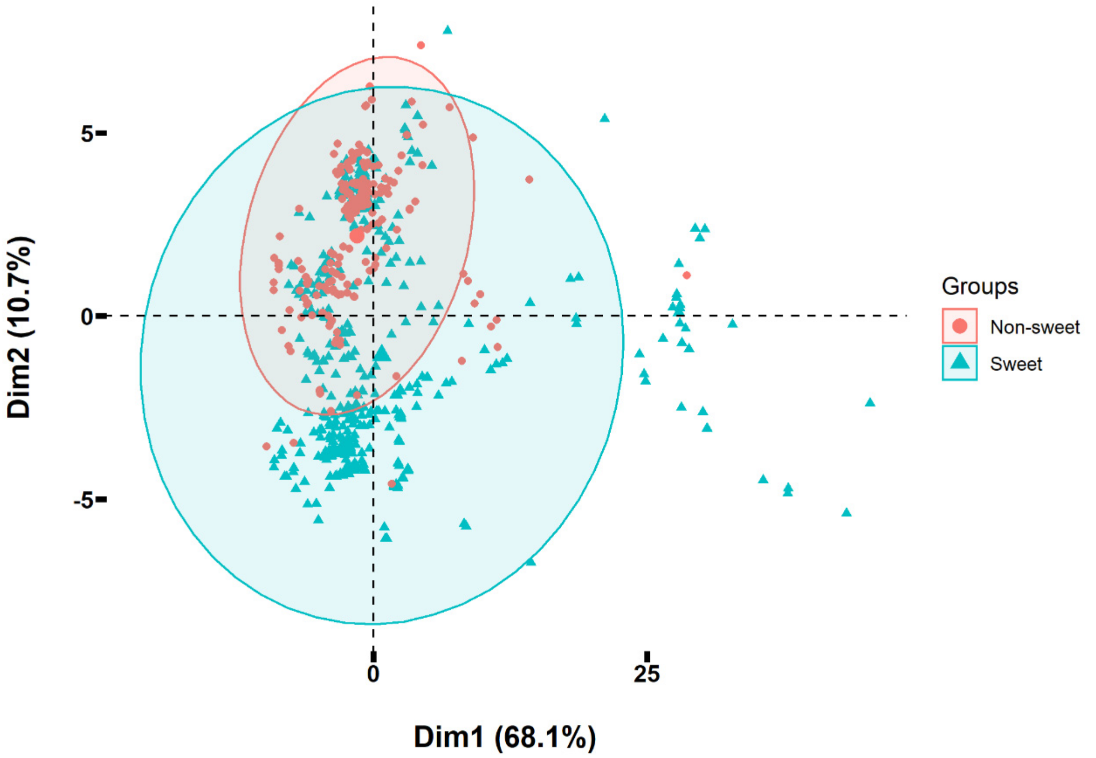

Figure S3

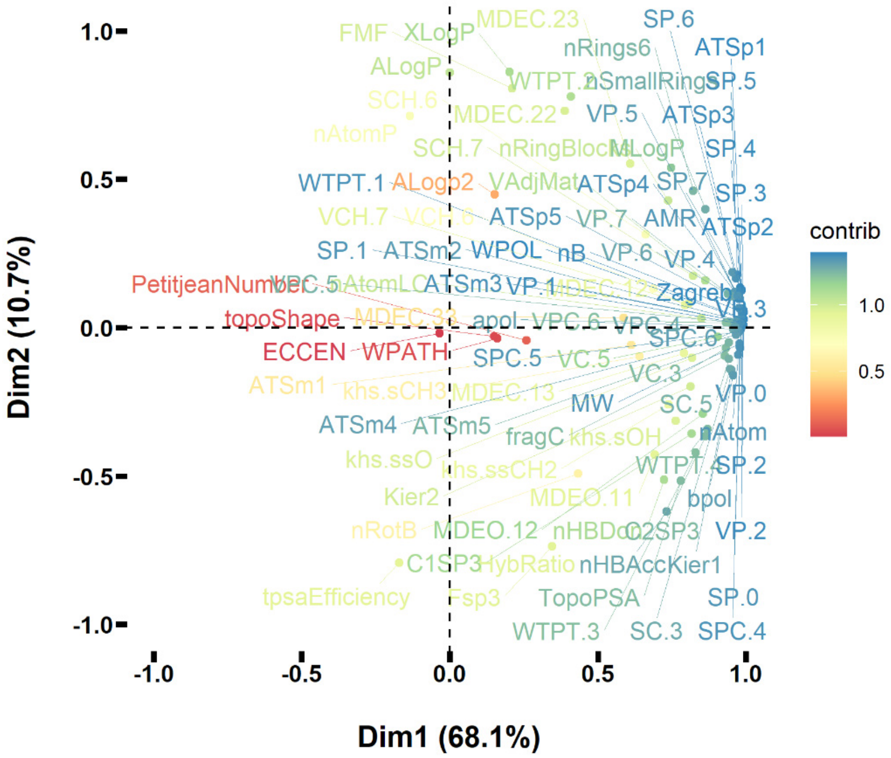

Figure S4

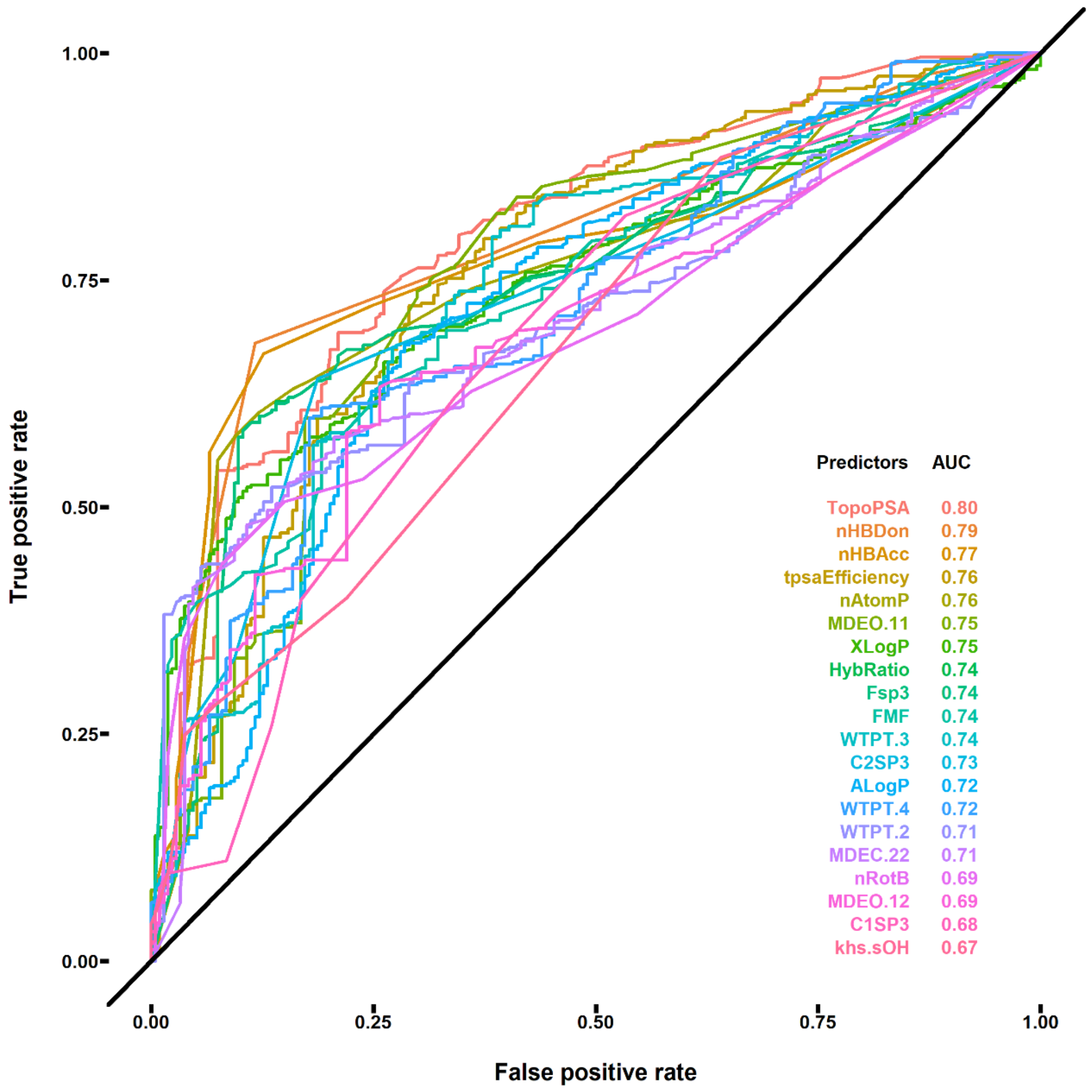
1.00
0.75
0.50
0.25
0.00
True positive rate
0.00
0.25
0.50
0.75
1.00
False positive rate
Predictors AUC
TopoPSA 0.80
nHBDon 0.79
nHBAcc 0.77
tpsaEfficiency 0.76
nAtomP 0.76
MDEO.11 0.75
XLogP 0.75
HybRatio 0.74
Fsp3 0.74
FMF 0.74
WTPT.3 0.74
C2SP3 0.73
ALogP 0.72
WTPT.4 0.72
WTPT.2 0.71
MDEC.22 0.71
nRotB 0.69
MDEO.12 0.69
C1SP3 0.68
khs.sOH 0.67

Figure S5

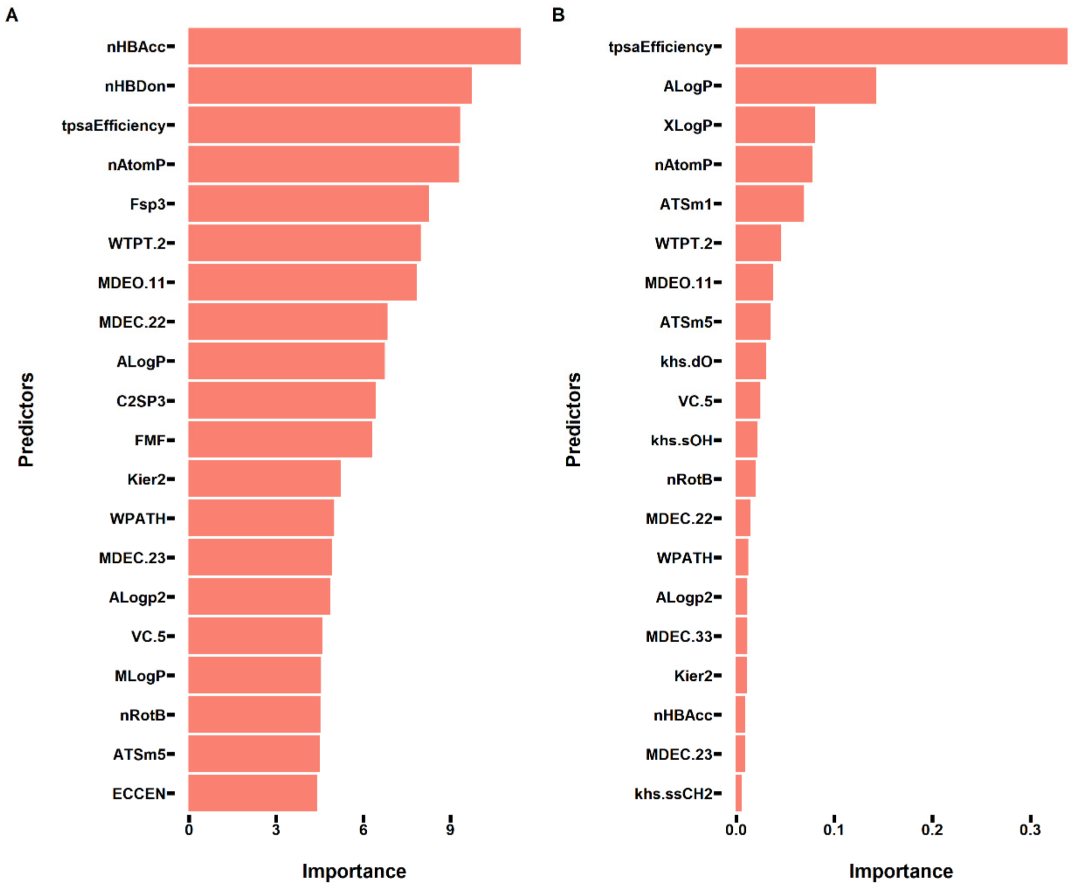
A
nHBAcc
nHBDon
tpsaEfficiency
nAtomP
Fsp3
WTPT.2
MDEO.11
MDEC.22
ALogP
C2SP3
FMF
Kier2
WPATH
MDEC.23
ALogp2
VC.5
MLogP
nRotB
ATSm5
ECCEN
Predictors
0
3
6
9
Importance
B
tpsaEfficiency
ALogP
XLogP
nAtomP
ATSm1
WTPT.2
MDEO.11
ATSm5
khs.dO
VC.5
khs.sOH
nRotB
MDEC.22
WPATH
ALogp2
MDEC.33
Kier2
nHBAcc
MDEC.23
khs.ssCH2
Predictors
0.0
0.1
0.2
0.3
Importance

Figure S6

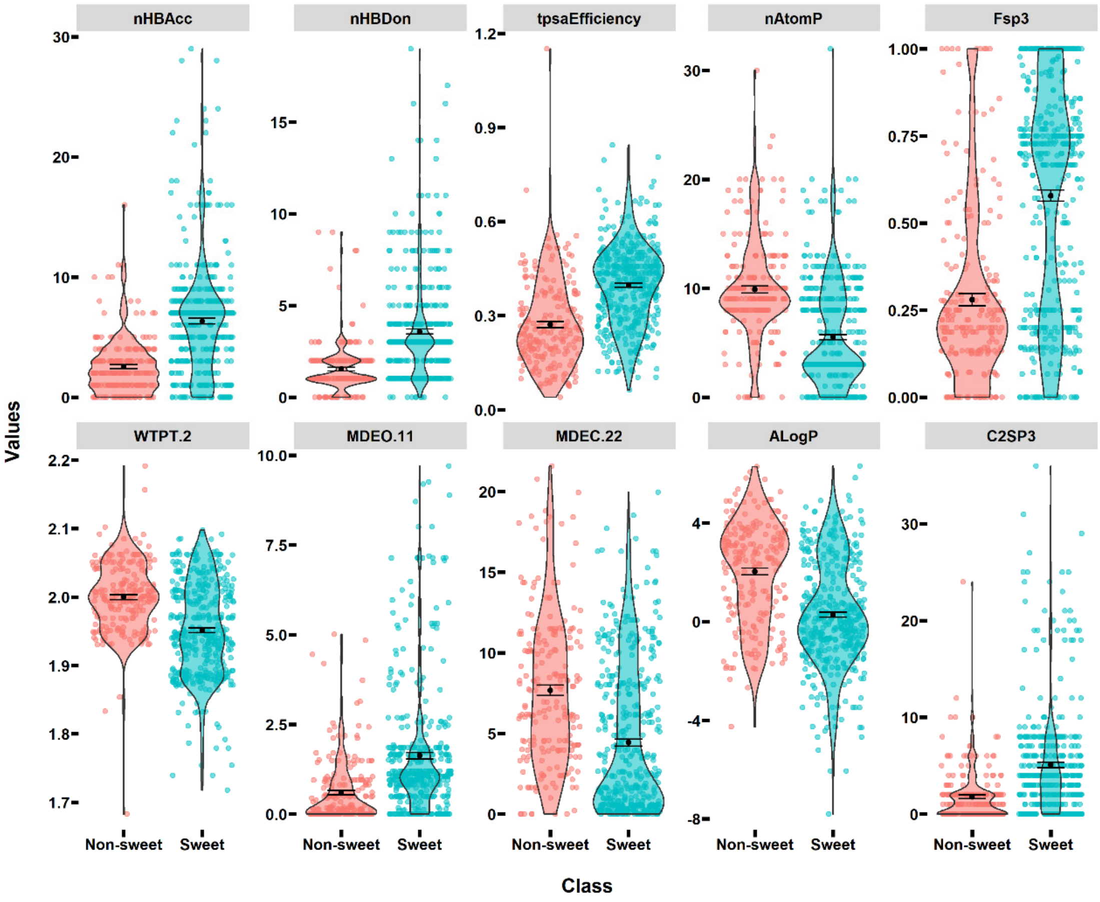
nHBAcc
nHBDon
tpsaEfficiency
nAtomP
Fsp3
WTPT.2
MDEO.11
MDEC.22
ALogP
C2SP3
Values
Non-sweet
Sweet
Class

Figure S7

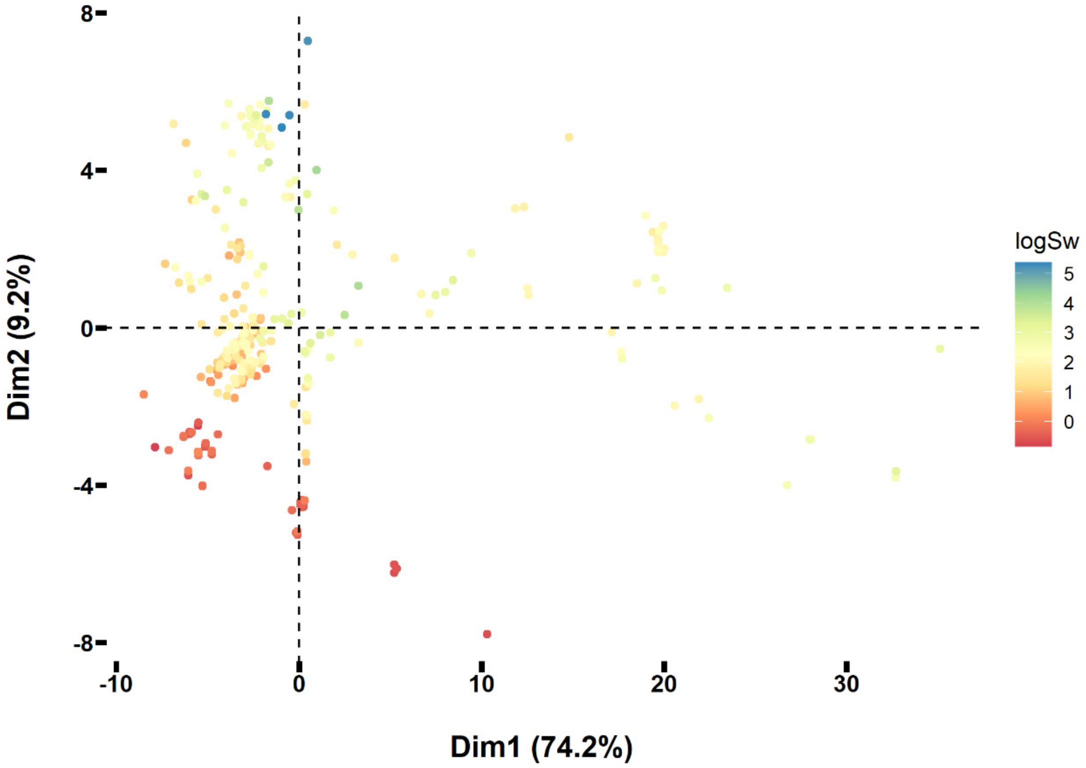

Figure S8

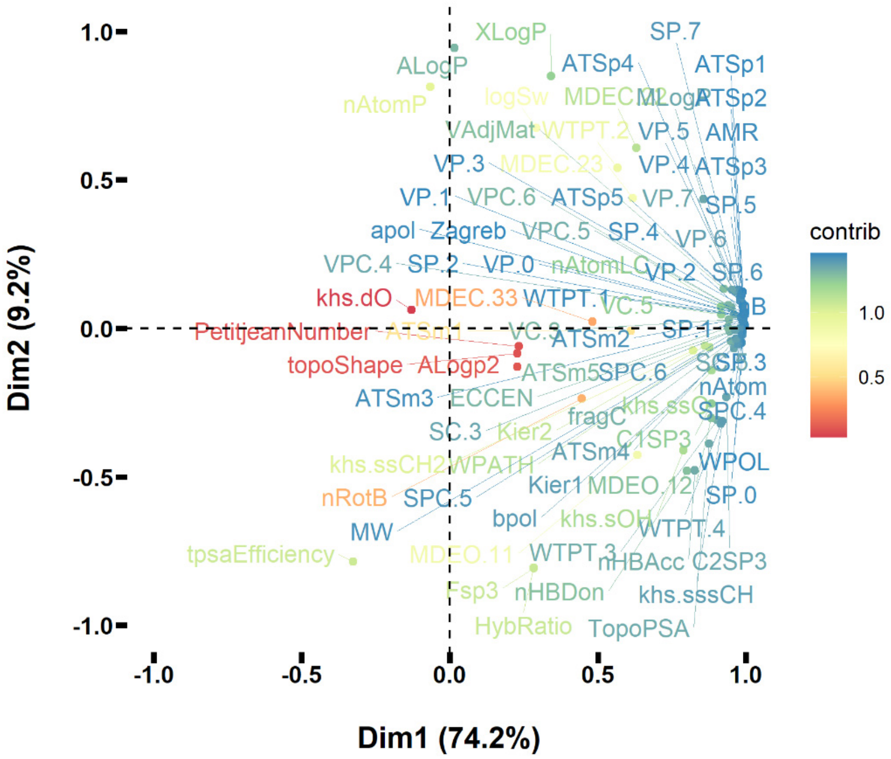
Dim1 (74.2%)
Dim2 (9.2%)
contrib
1.0
0.5
-1.0
-0.5
0.0
0.5
1.0
XLogP
SP.7
ALogP
ATSp4
ATSp1
nAtomP
ATSp2
AMR
ATSp3
VP.5
VP.4
VP.7
SP.5
VP.6
SP.6
VP.2
SP.1
SP.3
nAtom
SPC.4
WPOL
SP.0
WTPT.4
C2SP3
khs.sssCH
TopoPSA
HybRatio
nHBDon
nHBAcc
Fsp3
WTPT.3
MDEO.11
tpsaEfficiency
MW
bpol
khs.sOH
Kier1
MDEO.12
nRotB
SPC.5
khs.ssCH2
WPATH
ATSm4
C1SP3
SC.3
Kier2
fragC
ATSm3
ECCEN
topoShape
ALogp2
ATSm5
SPC.6
PetitjeanNumber
ATSm2
khs.dO
MDEC.33
WTPT.1
VC.5
VPC.4
SP.2
VP.0
apol
Zagreb
VPC.5
SP.4
VP.1
VPC.6
ATSp5
VP.3
MDEC.23
VAdjMat
WTPT.2
logSw
MDEC.22
MLogP

Figure S9

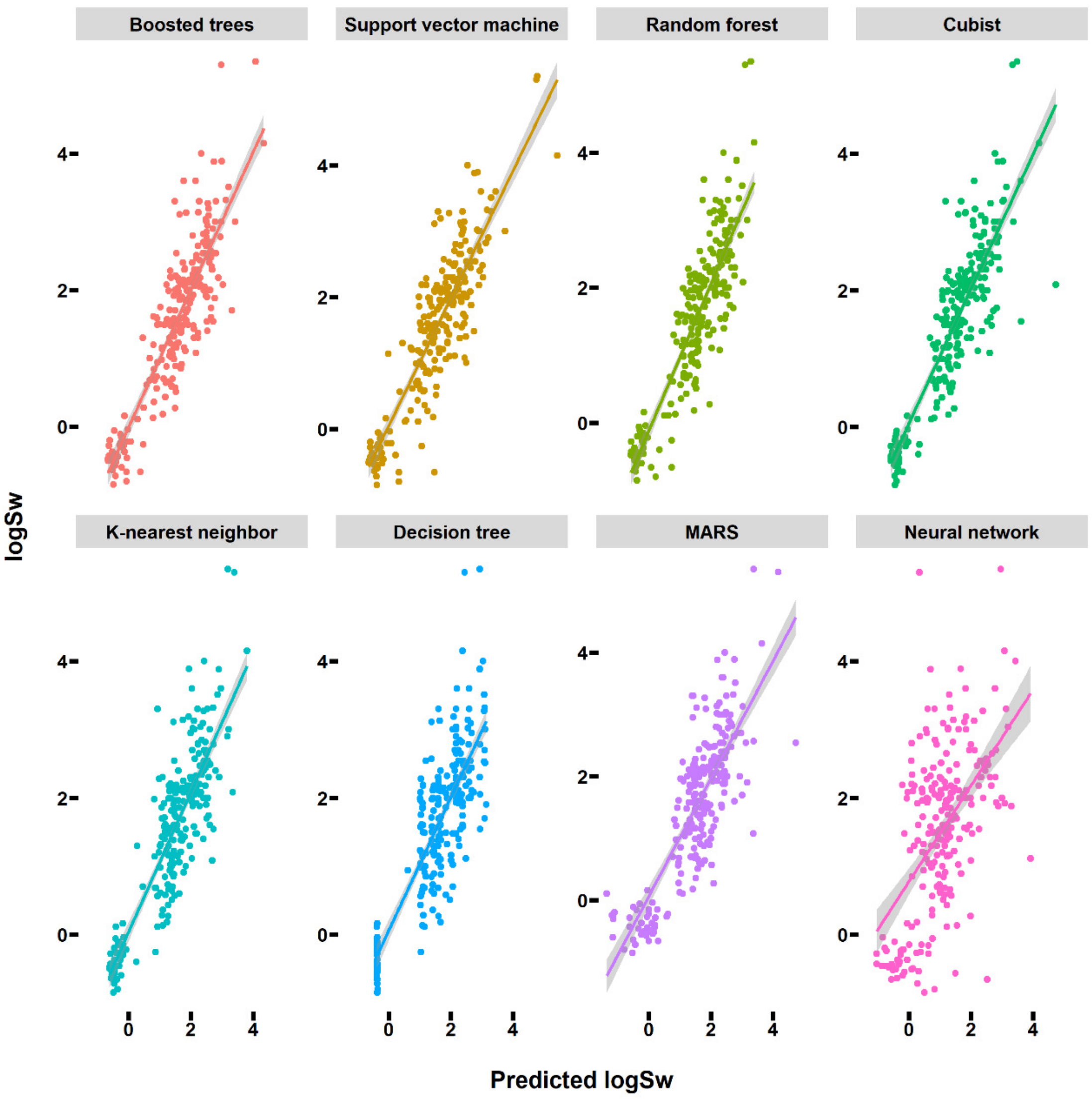

Boosted trees
Support vector machine
Random forest
Cubist
K-nearest neighbor
Decision tree
MARS
Neural network
logSw
Predicted logSw
0
2
4

Figure S10

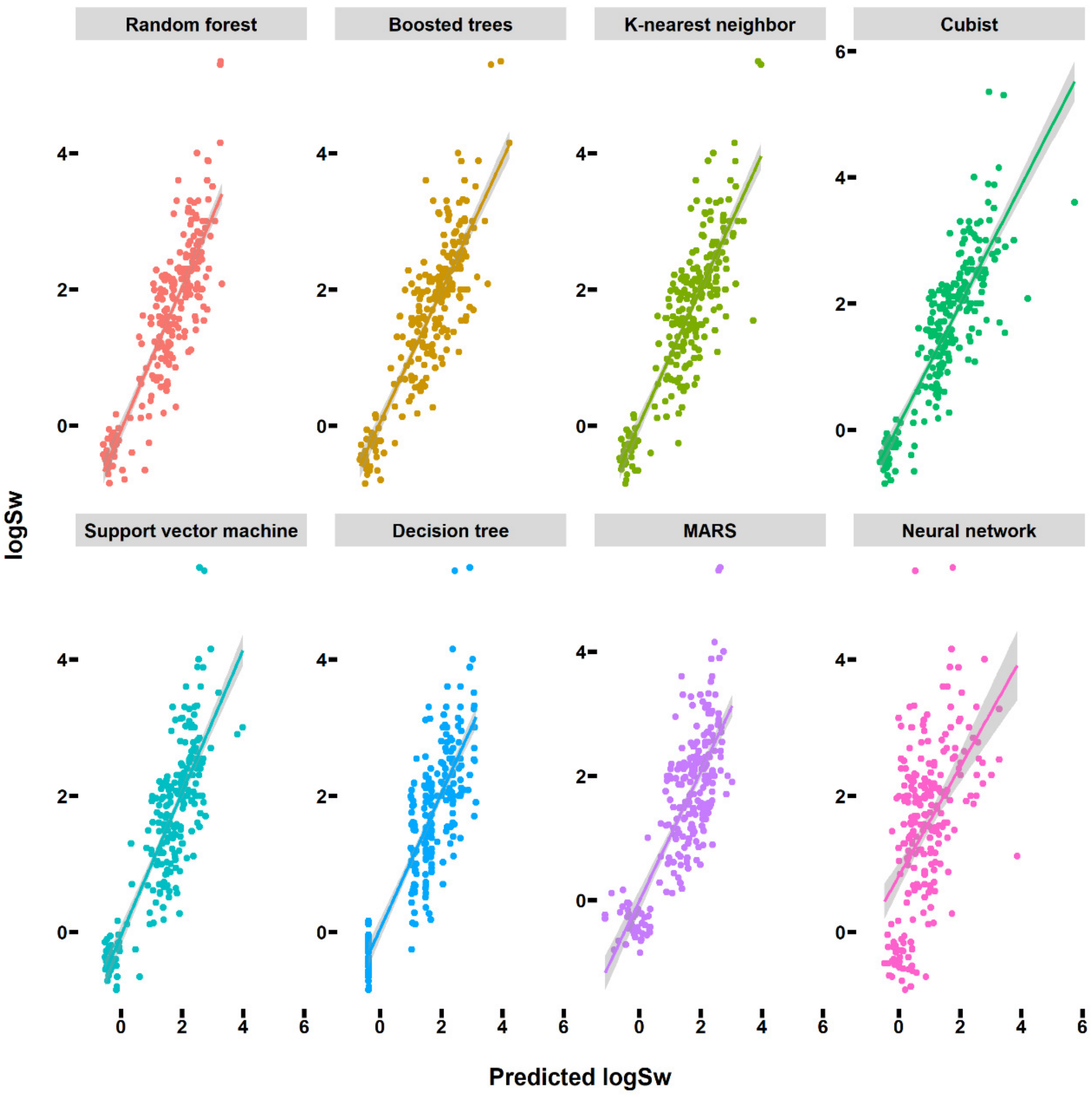

Random forest
Boosted trees
K-nearest neighbor
Cubist
Support vector machine
Decision tree
MARS
Neural network
logSw
Predicted logSw
0
2
4
6

Figure S11

A

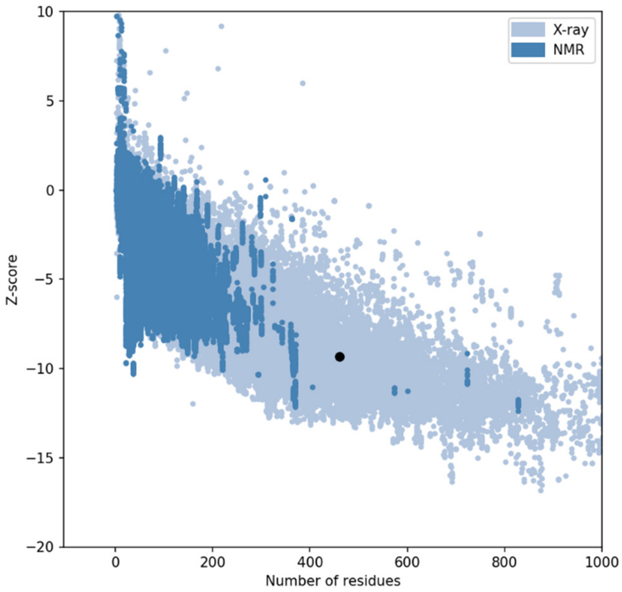

B

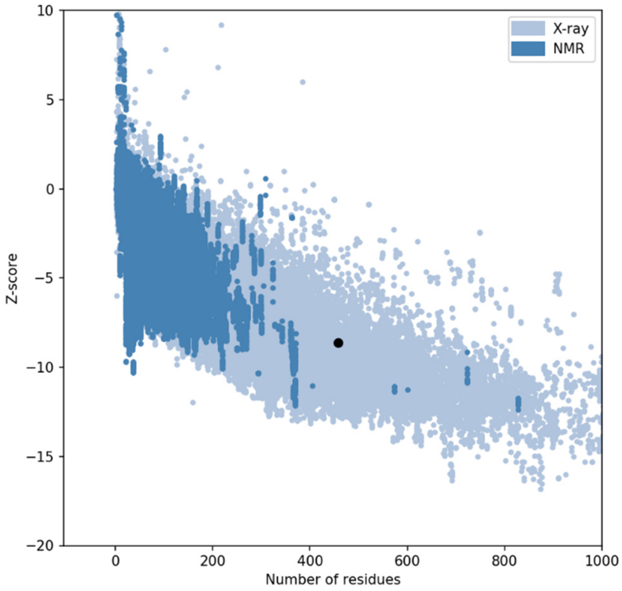

Figure S12
